# Aquaporin-4 Is Downregulated in the Basolateral Membrane of Ileum Epithelial Cells during Enterotoxigenic *Escherichia coli*-Induced Diarrhea in Mice

**DOI:** 10.3389/fmicb.2017.02655

**Published:** 2018-01-09

**Authors:** Di Zhang, Longfei Yang, Weiheng Su, Yuan Zhao, Xin Ma, Haizhu Zhou, Bo Xu, Kaiqi Zhang, Hongxia Ma

**Affiliations:** ^1^Department of Basic Veterinary Medicine, College of Animal Science and Technology, Jilin Agricultural University, Changchun, China; ^2^Jilin Provincial Key Laboratory on Molecular and Chemical Genetic, The Second Hospital of Jilin University, Changchun, China; ^3^National Engineering Laboratory for AIDS Vaccine, School of Life Science, Jilin University, Changchun, China; ^4^The Key Laboratory of Animal Production, Product Quality and Security, Ministry of Education, Jilin Agricultural University, Changchun, China

**Keywords:** aquaporin-4, diarrhea, enterotoxigenic *Escherichia coli*, ileum, mice

## Abstract

Enterotoxigenic *Escherichia coli* (ETEC) are opportunistic pathogens that colonize the small intestine, produce enterotoxins and induce diarrhea. Some aquaporins (AQPs), such as AQP3 and AQP8, have been reported to participate in diarrhea by decreasing cellular influx in the gastrointestinal (GI) tract. AQP4 is another important water channel in the GI tract, but its role in ETEC-induced diarrhea has not been reported. Here, we demonstrated the potential roles of AQP4 in ETEC-induced diarrhea. Reverse transcription-polymerase chain reaction (RT-PCR) and western blotting showed that AQP4 was expressed in the mouse ileum, but not in the duodenum or jejunum while immunohistochemical staining showed that AQP4 localized to the basolateral membrane of ileum epithelial cells. Using an ETEC-induced mice diarrhea model, we demonstrated that both AQP4 mRNA level and the AQP4 protein level in the ileum decreased gradually over a time course of 7 days. These results suggest that AQP4 plays a role in the pathogenesis of ETEC-induced diarrhea by mediating water transport.

## Introduction

The gastrointestinal (GI) tract transports more than 9 L of fluids on a daily basis by both absorptive and secretory processes including liquid in the diet (2 L) and intestinal secretions (7 L) (Masyuk et al., 2002). In addition, a small volume that is not absorbed (100–200 mL) is excreted in feces (Laforenza, 2012). Apparently, a disturbance in the absorptive mechanism can lead to excessive fluid loss from the GI tract and result in diarrhea. Aquaporins (AQPs) play important roles in transcellular water transport, and they are involved in diseases that are characterized by alterations in water transport, such as diarrhea (Zhu et al., 2016). Currently, AQP1, 3, 7, 9, 10, and 11 have been identified in the intestine of humans and rats, but their roles in diarrhea have not been fully explored (Hamabata et al., 2002; Laforenza et al., 2010). AQP4 is another important water channel in the GI tract, and it has been shown to be expressed and localized in the small intestine. Sakai et al. reported that the gene expression levels of AQP4 and AQP8 in the colon decreased significantly in 5-fluorouracil-induced diarrhea mice (Sakai et al., 2014), and AQP4 and AQP8 protein expression decreased significantly in the colon of rotavirus-induced diarrhea mice (Cao et al., 2014). Huang et al. reported that AQP4 mRNA and AQP4 protein level was downregulated in rotavirus infected Caco-2 cells (Huang et al., 2015).

Enterotoxigenic *Escherichia coli* (ETEC) strains are recognized as one of the major causative agents of dehydrating diarrhea in children in developing countries (Fleckenstein et al., 2010). ETEC can also cause diarrhea in newborn calves and in suckling or recently weaned piglet (Loos et al., 2012). ETEC induced diarrhea is caused by the action of toxic proteins known as enterotoxins. ETEC secretes at least one of two types of enterotoxins known as heat-labile and heat-stable enterotoxins, which increase the intracellular levels of cyclic nucleotides, resulting in the activation of the apical cystic fibrosis transmembrane regulator and, hence, promote Cl^−^ secretion (Bruins et al., 2006; Dubreuil, 2012). Overall, an osmotically driven increase in the permeation of water and electrolytes leads to fluid accumulation in the intestine. Although much is known about the role of ion channels in ETEC-induced diarrhea, insufficient attention has been paid to the pathways of AQP regulated water movement.

In this study, we examined whether there is a correlation between altered AQP4 expression and ETEC-induced diarrhea. The expression and localization of AQP4 were examined in various locations of the small intestine, and the potential alterations of AQP4 expression in the small intestine were analyzed in an ETEC-induced diarrhea mouse model.

## Materials and methods

### Animals

Transgenic knockout mice deficient in the AQP4 protein were provided by Dr. Tonghui Ma (Dalian Medical University, Dalian, China) (Ma et al., 1997). Studies were performed in age-matched wild-type and AQP4 knockout mice with a CD1 background. Mice were maintained in a specific-pathogen-free room kept at a constant temperature (22 ± 2°C) and humidity (55% ± 5%) on a 12 h light/dark cycle. All experiments had been approved by the Animal Care Committee at Jilin Agricultural University.

### Reverse transcription-polymerase chain reaction (RT-PCR)

Total RNA was extracted from small intestines using the RNeasy Micro Kit (Takara, Shiga, Japan). cDNA was generated from 2 μg of total RNA using the SuperScript First-strand Synthesis System (Invitrogen, Carlsbad, CA, USA). The resulting cDNA was used as the template with primers (sense, 5′-TGCCAGCTGTGATTCCAAACG-3′; and antisense, 5′-GCCTTCAGTGCTGTCCTCTAG-3′) flanking a 469-bp fragment of the AQP4 coding sequence. PCR products were analyzed subsequently by agarose gel electrophoresis.

### Western blotting

The freshly isolated small intestine was dissolved in lysis buffer (20 mM Tris, pH 7.5, 150 mM NaCl, 5 mM ethylenediaminetetraacetic acid, 1% Triton X-100, and 1 mM phenylmethylsulfonyl fluoride). Twenty micrograms of proteins were loaded into each lane of a sodium dodecyl sulfate−10% polyacrylamide gel, separated by electrophoresis and transferred to a polyvinylidene fluoride membrane. After blocking with 5% (w/v) nonfat milk for 30 min and washing with Tris-buffered saline containing Tween 20 (20 mM Tris pH 7.6, 0.2 M NaCl, and 0.1% Tween 20), the membrane was incubated with a rabbit anti-AQP4 polyclonal antibody (Sigma-Aldrich, St. Louis, MO, USA, 1:300 dilution) and washed three times. Then, the membrane was incubated with a goat anti-rabbit IgG antibody that was conjugated to horseradish peroxidase (HRP) (Sigma-Aldrich, 1:5,000 dilution). The secondary antibody was detected using an enhanced chemiluminescence kit (Amersham, Little Chalfont, UK).

### Immunohistochemistry

Mice were euthanized, and the small intestine was isolated, fixed with 4% paraformaldehyde, and embedded with paraffin. Continuous sections of the intestine were prepared in 3-μm widths following standard procedures. The sections were blocked using 5% goat serum, followed by incubation with a polyclonal anti-AQP4 antibody (Sigma-Aldrich, 1:200 dilution) in 0.1 M phosphate-buffered saline (PBS), 1% bovine serum albumin, and 0.5% Triton X-100 at room temperature for 1 h. After extensive rinsing with PBS, the sections were treated with an HRP-conjugated sheep anti-rabbit antibody for 1 h at room temperature. HRP activity was detected by reaction with diaminobenzidine.

### Induction of a diarrhea mouse model

ETEC strain O78: K88 (E44815) was obtained from the China Veterinary Culture Collection Center (Beijing, China). This ETEC strain was identified by PCR assays for the detection of genes encoding for enterotoxins, including heat-labile (LT) and heat-stable A and B (STa and STb) enterotoxins. The primers are: LT sense, CCGGTATTACAGAAATCTGA, antisense, GTGCATGATGAATCCAGGGT (product size, 272 bp); STa sense, CCGTGAAACAACATGACG, antisense, TGGAGCACAGGCAGGATT (product size, 168 bp); STb sense, GCAATAAGGTTGAGGTGAT, antisense, GCCTGCAGTGAGAAATGGAC (product size, 135 bp). Expression of STa gene was validated in the ETEC strain (**Figure 2A**). After hydration, the bacteria were cultured in Luria—Bertani broth medium at 37°C for 12 h until the absorbance at 600 nm reached 0.1 [an absorbance at 600 nm of 1 corresponds to 1 × 10^10^ colony-forming units (CFU)/mL] as determined by an ND-1000 UV-vis spectrophotometer (Thermo Fisher Scientific, Waltham, USA). The resulting cell density was estimated to be 1 × 10^9^ CFU/mL.

The diarrhea mouse model was established following protocols that were described previously (Deng et al., 2015). Briefly, male CD1 mice (8–9 weeks) were used to monitor diarrhea induced by ETEC suspensions (1 × 10^9^ CFU/mL, 3 mL) intragastrical administration. After acclimatization for 3 days and subsequent fasting for 6 h, the mice were assigned randomly into two groups: the control (PBS) group and the model group (diarrhea group). The control group received PBS, while the model group received one intragastric administration of an ETEC suspension in PBS. Body weights of the mice were recorded daily, and a diarrhea score was determined at 0–7 days after ETEC administration. Animals were sacrificed, and their ilea were isolated when the diarrhea model was successfully established at 0, 1, 3, 5, and 7 days after ETEC administration, and the total RNA and protein were extract immediately in order to avoid degradation.

### Diarrhea assessment

A diarrhea score was determined for each mouse following standard protocols (Sakai et al., 2013). The severity of ETEC-induced diarrhea was scaled as follows: (0) normal stool; (1) stool; (2) soft and slightly wet stool; (3) wet and unformed stool with moderate perianal staining of the coat; and (4) watery stool with severe perianal staining of the coat.

### Quantitative real-time PCR

Total RNA was extracted from the jejunum with guanidium-phenol chloroform using the TRIzol reagent (Sigma-Aldrich). cDNA was prepared from 1.0 μg of RNA with the PrimeScript RT reagent Kit with gDNA Eraser (Takara). Two microliters of the resultant reaction mixture were used as the template for a subsequent 10-μL PCR that also contained 50 nM of the forward and reverse primers and Fast SYBER Green Mastermix (Applied Biosystems, Foster City, CA, USA). The primers for AQP4 were: forward, 5′-CTGGAGCCAGCATGAATCCAG-3′; and reverse, 5′-TTCTTCTCTTCCCACGGTCA-3′. β-actin expression was used as a control, and the β-actin gene was amplified with the following primers: forward, 5′-CCACCATGTACCCAGGCATT-3′; and reverse, 5′-GGACTCATCGTACTCCTGC-3′. The ratio of AQP4 expression to β-actin expression was analyzed using the 2^−ΔΔCT^ method (CT is the cycle threshold), where −ΔΔCT = −(ΔCT of the experimental group −ΔCT of the control group) and ΔCT = CT of the samples − CT of β-actin.

### Enzyme-linked immunosorbent assay (ELISA)

The concentration of AQP4 in protein extracts of the ileum was measured with a sandwich ELISA method using the Mouse AQP4 ELISA Kit (Elabscience, Bethesda, MD, USA) following the manufacturer's protocols. The final concentration of the AQP4 was normalized according to a reference standard.

### Statistical analysis

Data were presented as means ± standard errors (SDs). An unpaired Student's *t*-test was used to assess the statistical significance of differences between the two groups.

## Results

### Aquaporin 4 is expressed and localizes in the basolateral membrane of deep crypt epithelia in mouse ileum

**Figure 1:**
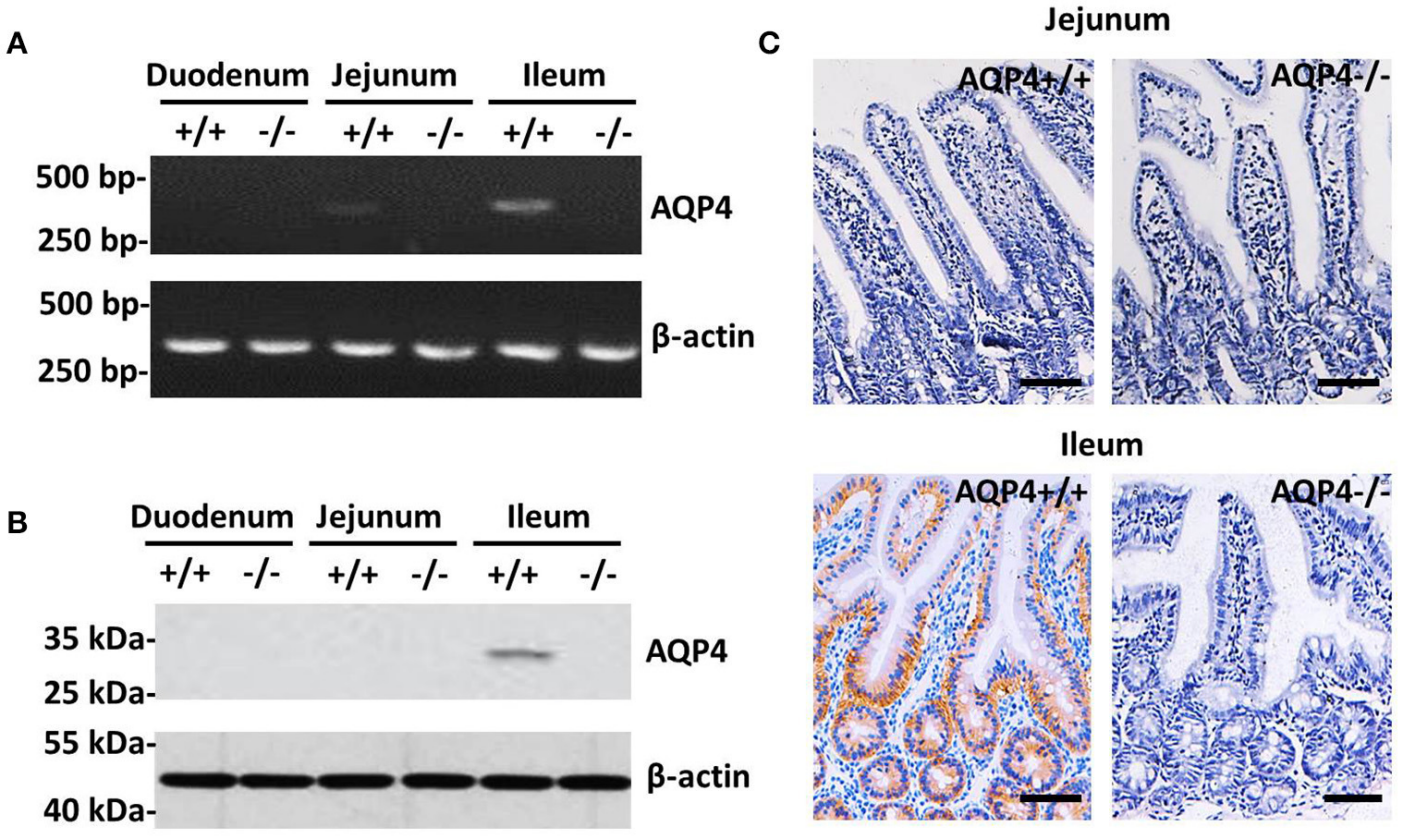
Expression and localization of AQP4 in the small intestine of mice. **(A)** RT-PCR detection of AQP4 mRNA from the duodenum, jejunum, and ileum. **(B)** Western blotting detection of AQP4 from the duodenum, jejunum, and ileum. **(C)** Immunohistochemical localization of AQP4 in the basolateral membrane of ileum epithelial cells, but not in the jejunum. Bars = 100 μm.

The expression of AQP4 was detected in the ileum, but not in the jejunum or duodenum. Using RT-PCR, AQP4 mRNA was detected in the ileum in a significant amount and in the jejunum at a barely detectable level, but not in the duodenum of wild-type mice (Figure 1A). However, the AQP4 protein was identified by western blotting as a 34-kDa band only in the ileum of wild-type mice (Figure 1B), but not in the jejunum. To further localize AQP4, we performed an immunohistochemical analysis of the small intestine using polyclonal AQP4 antibodies, which showed that the AQP4 protein resided in the basolateral membrane of ileum epithelial cells, but not in the jejunum (Figure 1C). Collectively, we conclude that meaningful amount of AQP4 localizes only in the basolateral membrane of ileum epithelial cells of mice.

### Establishment of an ETEC-induced diarrhea mice model

**Figure 2:**
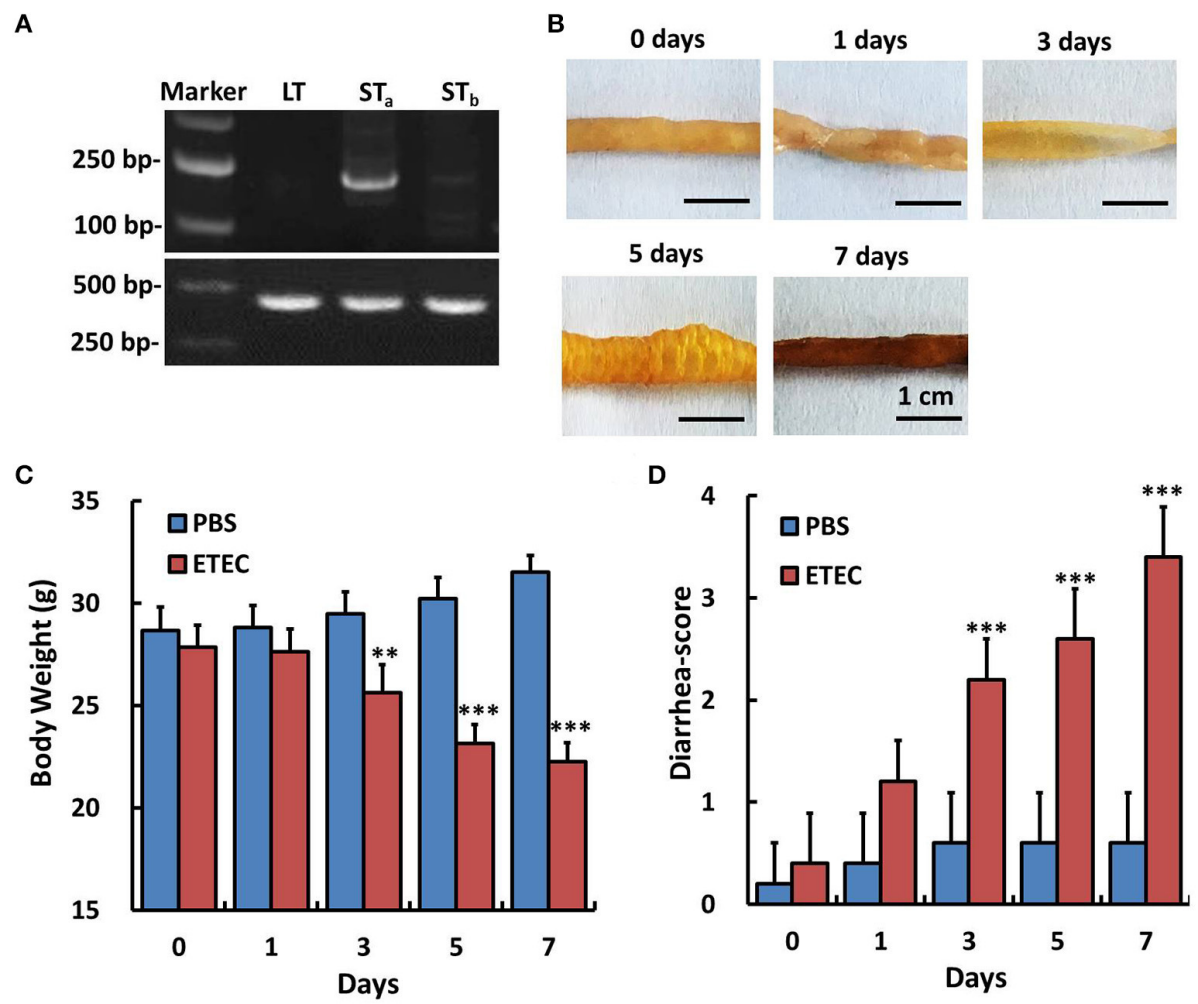
Detection of enterotoxin genes in the ETEC strain and the effect of ETEC administration on the body weight and diarrhea score of mice. The genes encoding heat-labile toxin (LT), heat-stable toxin A and B (STa, STb) of ETEC were amplified by PCR. We validated that STa gene was expressed in this ETEC strain. Experiments were performed immediately after treatment (0 day) and at 1, 3, 5, and 7 days after ETEC (1 × 10^9^ CFU/mL, 3 mL, intragastric) or PBS (control, intragastric) administration. **(A)** RT-PCR assay for the detection of enterotoxin genes in the Enterotoxigenic *Escherichia coli* strain. **(B)** Gross images of the ileum after ETEC administration. **(C)** Changes in body weight after ETEC administration. **(D)** Changes of the diarrhea score after ETEC administration. Each point represents the mean ± SD of 6–8 mice. ^**^p < 0.01, and ^***^p < 0.001 vs. the control (PBS) group.

An ETEC-induced diarrhea mice model was established and evaluated by the methods described in the previous literature (Deng et al., 2015). As shown in Figure 2B, at days 3 and 5, there was a significant accumulation of fluid in the intestinal lumen, and at day 7, hemorrhage of the small intestinal was observed. The body weight and the diarrhea scores in all the mice were observed and recorded during infection of ETEC for 7 days. The body weight of the mice decreased significantly from days 3 to 7 in the model group compared with the control (PBS) group (Figure 2C), while the diarrhea scores in the model group increased significantly from days 3 to 7 (Figure 2D).

### AQP4 mRNA and protein levels decrease in ETEC-induced diarrhea mice

**Figure 3:**
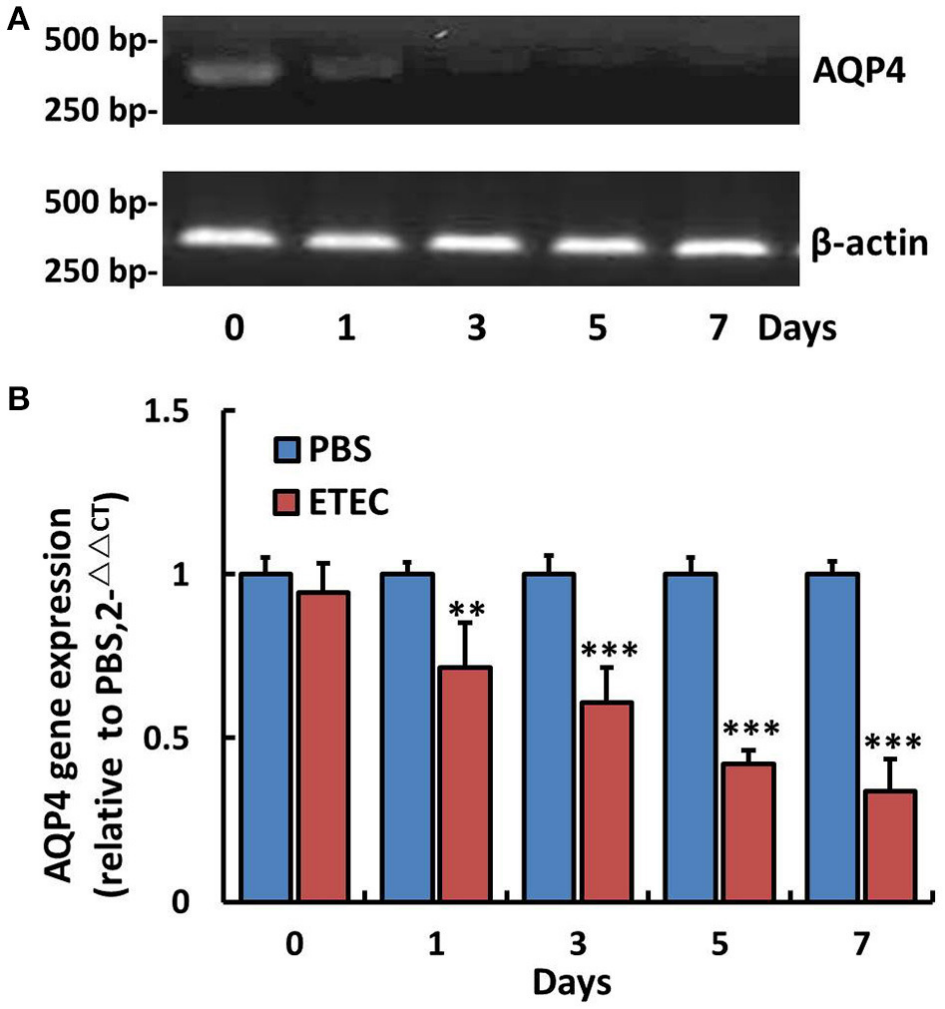
Changes in AQP4 gene expression in the ileum of mice after ETEC administration. Treatment with ETEC decreased AQP4 gene expression in the ileum of mice. **(A)** Expression of AQP4 mRNA as detected by agarose gel electrophoresis after RT-PCR. **(B)** Quantification of AQP4 mRNA expression by quantitative real-time PCR. Each column represents the mean ± SD of 6–8 mice. ^**^p < 0.01 and ^***^p < 0.001 vs. the control (PBS) group.

**Figure 4:**
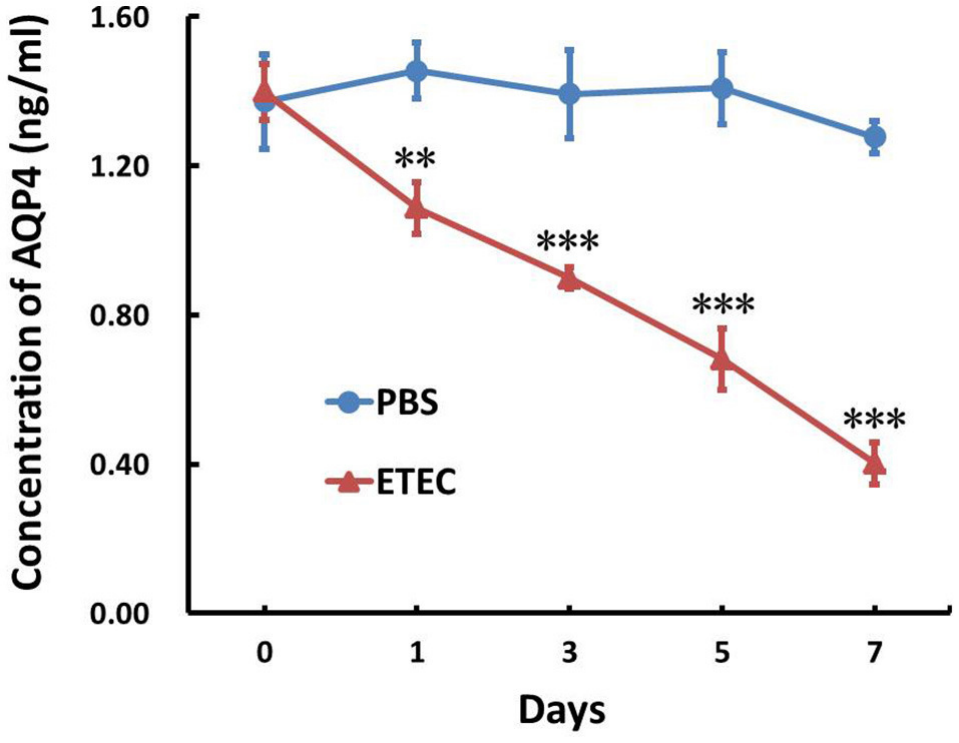
Effect of ETEC on the concentration of AQP4 in the ileum. Treatment with ETEC decreased the AQP4 protein concentration in the ileum of mice as determined by an ELISA. Each column represents the mean ± SD of 6–8 mice. ^**^p < 0.01, and ^***^p < 0.001 vs. the control (PBS) group.

The AQP4 mRNA level in the ileum was first evaluated by RT-PCR (Figure 3A) and subsequently quantified by real-time PCR (Figure 3B). As shown in Figure 3, it decreased significantly in a time-dependent manner over the 7-day course of infection. The association between AQP4 expression and ETEC diarrhea was confirmed by ELISA, which showed a time-dependent decrease of AQP4 expression after the administration of ETEC (Figure 4).

## Discussion

ETEC is a major cause of diarrhea in developing countries and severe cases cause life-threatening dehydration. In addition, diarrhea caused by ETEC is a significant threat to formed animals, e.g., calves and piglet (Dubreuil et al., 2016). ETEC strains causes diarrhea by colonizing to the epithelium of the small bowel and elaborating their enterotoxins to cause massive loss of fluid into the bowel lumen. Several studies have attempted to link diarrheal mechanisms with decreases in the levels of AQPs. Julian et al. reported that both AQP2 and AQP3 were absent from colonocyte membranes infected by enterohemorrhagic *Escherichia coli* and enteropathogenic *Escherichia coli* (Guttman et al., 2007). Consistently, Ikarashi et al. reported that the inhibition of AQP3 in the colon after the rectal administration of HgCl_2_ or CuSO_4_ may suppress water transport from the luminal side to the vascular side, leading to diarrhea (Ikarashi et al., 2012). Here, we found that the expression of AQP4 mRNA decreased significantly after 12–24 h of dextran sodium sulfate exposure and remained depressed throughout the treatment period in a mouse model of colitis (Hardin et al., 2004). Collectively, the expression of AQPs plays a significant role in ETEC-induced diarrhea.

ETEC is the leading cause of diarrhea in humans and farm animals (Nagy and Fekete, 2005; Qadri et al., 2005). In the present study, ETEC localized in the small intestine and induced the loss of an excessive amount of fluid most likely by secreting an enterotoxin. The excessive loss of water in ETEC-induced diarrhea is caused by the action of transport proteins in the intestinal lumen, such as the cystic fibrosis transmembrane regulator and calcium-activated potassium ion channels (Rosa et al., 2013). Recently, it was suggested that ETEC-induced diarrhea was also associated with defects in water transport, which were associated with reduced expression of the AQPs.

Traditionally, the diarrhea caused by ETEC was attributed to the direct fluid loss by the actions of two enterotoxins, heat-labile and heat-stable enterotoxins. Heat-stable toxin stimulates guanylate cyclase while heat-labile toxin stimulates adenylate cyclase to increase the intracellular levels of cGMP and cAMP to cause fluid loss in the lumen of small bowl (Dubreuil, 2012). This did not fully account for the strong water reabsorbing ability of small bowels, which regularly reabsorb ~7 L water per day through the actions of aquaporins. Here, we provide an additional mechanism of massive fluid loss, by destructing AQPs to prevent water reuptake in the epithelial cells. Consistently, it has been established that ETEC produces highly conserved proteases to destroy proteins of epithelial cells of host intestine (Lou et al., 2014). Thus, the degradation of AQPs in ETEC induced diarrhea could well be the actions of those proteases. In the current study, the presence of AQP4 in the GI tract clearly illustrated its role in fluid absorption. AQP4 was expressed in ileum epithelial cells of the small intestine, as detected by RT-PCR and western blotting (Figures 1A,B), and immunohistochemistry revealed that it localized to the basolateral membrane (Figure 1C). The unique expression pattern is similar to that reported in a previous study by Jiang et al., who showed that AQP4 was expressed widely in absorptive and glandular epithelial cells of the guinea pig small and large intestines (Jiang et al., 2014). Wang et al. reported that AQP4 localizes to the basolateral membrane of surface colonocytes and that the water content in defecated stool was higher in AQP4 null mice than in wild-type mice, suggesting that AQP4 may also play a role in colonic fluid absorption (Wang et al., 2000). As such, further studies are warranted for the detailed mechanisms of GI water transport by AQP4.

ETEC is usually not invasive and the preferred treatment of endemic ETEC is oral fluid replacement in the majority cases (IV fluid replacement in moderate to severe cases). Antibiotics are generally not needed except in severe cases, because their application will not significantly reduce the duration of diarrhea unless applied early in disease and is closely associated with drug resistance (Sarker et al., 2016). AQPs, shown in this study and other studies, as important factors that are destroyed during ETEC diarrhea are likely to be the protective factors for ETEC diarrhea (Zhang et al., 2017). As such, stabilization of AQPs could be a more effective treatment than fluid replacement for ETEC induced and potentially other diarrheas. One of the ways to stabilize AQPs during ETEC diarrhea is to destroy the recognition of AQPs by ETEC factors, which will require subsequent studies on the details mechanisms for the recognition and destruction of AQPs. In humans AQP4 appears to be expressed in brain and lung but not in the intestine (Song et al., 2017). In mice the gene appears to be preferentially transcribed in brain and colon (Verkman, 2002), while in rats AQP4 has been shown to be present in the basolateral small intestine (Koyama et al., 1999). Consistently, certain strains of ETEC preferentially localized in enterocytes of mouse ileal mucosa (Allen et al., 2006). Taken together, this could be why we observed very severe diarrhea and weight loss in ETEC challenged mice while such severe symptoms seem uncommon in human.

Aquaporins have long been suggested to be drug targets for diarrhea (Ikarashi et al., 2016). In colon, transcellular water transport using AQP4 in the mucosal epithelial cells is an important but non-dominant role that modulates the water content of feces (Jiang et al., 2014). Considering that colon is only responsible for 1.3 L of water transport per day while small intestine is responsible for 6.5 L of water transport, the water transport of AQP4 is likely to play a bigger role in the small intestine (Ma and Verkman, 1999). As such, the correlation of ETEC-induced diarrhea and disappearance of AQP4 is likely through water transport.

In conclusion, the current study showed that AQP4 was expressed in ileum epithelial cells and downregulated upon the administration of ETEC to mice. We propose that changes in the distribution of AQP4 plays a role in the pathogenesis of ETEC-induced diarrhea by mediating water transport. Further studies are required to establish the molecular mechanism connecting ETEC-induced diarrhea and AQP4 expression.

## Author contributions

DZ, WS, and HM: Conceived and designed the experiments; DZ, LY, KZ, YZ, HZ, BX, and XM: Performed the experiments; DZ and KZ: Analyzed the data; DZ and WS: Wrote the paper.

### Conflict of interest statement

The authors declare that the research was conducted in the absence of any commercial or financial relationships that could be construed as a potential conflict of interest.
